# Integration of imaging modalities in digital dental workflows - possibilities, limitations, and potential future developments

**DOI:** 10.1259/dmfr.20210268

**Published:** 2021-09-14

**Authors:** Sohaib Shujaat, Michael M. Bornstein, Jeffery B. Price, Reinhilde Jacobs

**Affiliations:** 1 Department of Imaging & Pathology, Faculty of Medicine, KU Leuven & Oral and Maxillofacial Surgery, University Hospitals Leuven, Leuven, Belgium; 2 Department of Oral Health & Medicine, University Center for Dental Medicine Basel UZB, University of Basel, Basel, Switzerland; 3 Department of Oncology and Diagnostic Sciences, University of Maryland, School of Dentistry, Baltimore, Maryland, USA; 4 Department of Dental Medicine, Karolinska Institutet, Stockholm, Sweden

**Keywords:** dentistry, computer simulation, workflow, digital image processing, cone-beam computed tomography

## Abstract

The digital workflow process follows different steps for all dental specialties. However, the main ingredient for the diagnosis, treatment planning and follow-up workflow recipes is the imaging chain. The steps in the imaging chain usually include all or at least some of the following modalities: cone-beam computed tomographic data acquisition, segmentation of the cone-beam computed tomography image, intraoral scanning, facial three-dimensional soft tissue capture and superimposition of all the images for the creation of a virtual augmented model. As a relevant clinical problem, the accumulation of error at each step of the chain might negatively influence the final outcome. For an efficient digital workflow, it is important to be aware of the existing challenges within the imaging chain. Furthermore, artificial intelligence-based strategies need to be integrated in the future to make the workflow more simplified, accurate and efficient.

## Introduction

When trawling through advancements in dental medicine, the classical steps in conventional workflows which were once considered as the norm, such as acquiring alginate impressions, treatment planning and template designing on gypsum-based models and relying on two-dimensional (2D) images of three-dimensional (3D) dentomaxillofacial structures, are constantly being superseded by more accurate and efficient digitized approaches.^1,2^ The term computer-aided design and computer-aided manufacturing (CAD-CAM) dentistry should not be confused with digital dentistry, where CAD-CAM only refers to the application of technological resources aiding clinicians to perform dentomaxillofacial procedures by virtual designing and 3D printing. Digital dentistry is a broader term and covers the inclusion of computer-based tools guidance for a wide variety of applications, such as, patient data collection, communication, diagnosis, treatment planning and follow-up assessment.^3^

The main developments in digital dentistry which have allowed most improvements in the workflows include low-dose high-resolution cone-beam computed tomography (CBCT) scanners, intraoral scanners (IOS), CAD-CAM software programs, medical 3D printers and dynamic navigation systems.^4^ This digitization in dental medicine has not just altered the functioning and thinking of dentists but also played an important role in improving the patients’ experience. It has had a strong impact specifically for specialties such as dental implantology, restorative dentistry, endodontics and orthodontics, by constantly aiming for and ideally delivering a more accurate treatment with improved outcomes, a reduced complication rate and better patient monitoring.^5,6^ Also, further advancements are being made to simplify these digital workflows.^7–9^

The implementation of digital workflows in conventional orthodontics has allowed fabrication of various orthodontic appliances through 3D printing, virtual planning of brackets and the possibility to review the predicted outcomes beforehand.^10^ In restorative and prosthetic dentistry, it is now possible to fabricate and provide patients with the crown and/or bridge restoration and denture through the digital workflow and additive manufacturing with a reduction in the number of visits, which once required multiple appointments using a traditional workflow.^11–13^ In implantology, endodontics and oral and maxillofacial surgery, digital designing of guides and templates have allowed for more precise provision of the treatment by enabling ideally a flapless insertion of dental implants.^14–16^ Furthermore, application of the dynamic navigation systems in the digital chain have had a strong impact in the digital world by providing clinicians a virtual environment and real-time guidance for diagnostics, treatment planning and performing both simple and complex dental procedures with a higher accuracy and precision compared to the freehand or even static guided approaches.^17,18^

The digital workflow process in all dental specialties follows different steps. However, the most commonly shared main component between most of the clinical diagnostic, treatment planning and follow-up workflow recipes is the imaging chain for the creation of a virtual augmented model.^19,20^ Clinically, it is pivotal to have a precise imaging chain for the generation of a model with sufficient details of the anatomical structures in question, enabling practitioners to accurately deliver the virtual planning in reality. An inadequate virtual model might lead to an inappropriate patient assessment, flawed treatment planning and unsatisfactory clinical outcomes. Therefore, the aim of this review was to provide an evidence-based overview of the possibilities and pitfalls associated with the integration of CBCT scanning, as well as intraoral and facial soft tissue imaging for the creation of a virtual model. Furthermore, recommendations for improving the existing imaging workflows will be provided.

### CBCT image acquisition

The first step, which paved the way towards digitizing dental workflows was the introduction of CT scanners. With the popularization of medical-grade helical CT scanners, early applications for dental medicine became available, which included utilization of the cross-sectional images for pre-operative assessment of the jaws for endosseous implant surgery.^21–24^ Later, 3D planning software programs were introduced allowing reformatting of the CT-based cross-sectional images for the visualization of bone height/width, virtual placement of dental implants and 3D simulation of the surgical procedures.^25^ However, the wider implementation of CT scanners for regular dental applications was impossible due to the large size and cost of the machines, high radiation exposure and risks that outweighed potential benefits. To overcome these limitations, it was in 1998, when the first CBCT device, the NewTom 9000 (Quantitative Radiology, Verona, Italy), was introduced in the field of dental medicine.^26^ Although it offered lower radiation exposure, the image quality was still not up to par to that of a CT scanner. Since then, CBCT devices have undergone multiple modifications and are now able to offer low-dose high quality images with the availability of limited field of view (FOV). The constant developments in hardware and software programs with cutting-edge reconstruction algorithms have greatly contributed towards reduction in the radiation dose using 3D imaging. However, this dose reduction is also associated with a suboptimal image quality, which might negatively influence the 3D dentomaxillofacial treatment planning.^27–29^

The most troublesome aspect in achieving an optimal accuracy of the digital workflow is the actual image acquisition, which can influence the later steps and the final outcome. Currently, approximately 279 CBCT devices exist in the market with variable technical parameters.^30^ The majority of the devices available offer a variable radiation dose, ranging between 10 and 1000 μSv, which corresponds to 2–200 panoramic radiographs.^31–33^ A limited amount of information is provided by most of the companies pertaining to the technical characteristics of the CBCT devices, such as the effective radiation dose range.^34^ Furthermore, a wide difference exists amongst and even within the same CBCT devices in relation to the dose depending on the technical settings, where some devices are able to provide a 3D image at a lower dose compared to others which offer a radiation level equivalent to that of a medical CT.^30^ Thereby, it is recommended that companies provide radiologists with all the technical parameters, so further optimization of the settings can be achieved, allowing a balance between a low radiation exposure and an optimal image quality for reconstructing an accurate virtual model. In a dental practice, an accurate virtual model is vital for establishing a definitive and objective treatment plan and follow-up assessment when using a digital workflow, which might in turn improve patient care. For instance, creating guides for a precise dental implant insertion and endodontic treatment, design and production of dental restorations, virtual orthodontic and surgical planning, transfer of planning in combined orthodontic–orthognathic and oral oncology procedures and assessing treatment outcomes by superimposing virtual models at different time points.^5^

Amongst the exposure parameters such as tube voltage (kV) and tube current (mA), most devices offer adjustable settings to alter these parameters depending on the task at hand while in some they are fixed.^30^ It is important to realize that these features can drastically alter radiation dose exposure as well as image quality. Thus, it only makes sense that a radiologist should have a manual control over these parameters to achieve a high quality image while following the ALADAIP (as low as diagnostically acceptable being indication-oriented and patient-specific) principles.^35^

Even though kV and mA are adjustable in most devices, still no clinical evidence exists related to the optimal scanning settings for reducing the influence of streak metal artifacts,^36^ which needs to be explored in future studies. Another important parameter is the FOV, where agreement exists that ideally a smaller FOV should be selected to reduce radiation dose exposure to the patient.^37^ Although a smaller FOV is optimal for general diagnosis, a larger FOV might be indicated for using CBCT during the digital workflow related to dental implant treatment planning, since these cases require increased numbers of data points for an accurate merging, superimposition and stable placement of surgical guides. In addition, cases involving oral pathology might also require visualization through a larger FOV to include also areas of healthy bone surrounding the lesion.^5^

Finally, the voxel size selection also influences the image quality and radiation dose. A smaller voxel size offers higher image quality with increased radiation exposure and vice versa. Some specific workflows in which small structures such as root canals, root fracture/resorption or periodontal tissue need to be visualized optimally for treatment planning, a smaller voxel size would be more feasible.^33^

All the aforementioned acquisition parameters have the ability to influence the accuracy of later steps for generating a virtual model such as segmentation and registration, which in turn can lead to a suboptimal treatment planning and outcome. Further research and formulation of guidelines is required to optimize these parameters in relation to the digital workflows. As the prior guidelines have been laid based solely on the diagnostic quality of the images without characterizing the influence of image quality on the accuracy of a digital workflow.^35^ The guidelines should focus towards achieving a balance between low radiation dose and optimal image quality for the creation of a virtual model.

### Segmentation

The second most critical step in the digital workflow consists of image segmentation for generating a 3D model of the anatomical region of interest. These 3D surface-rendered volumetric models are an integral part of orthodontic, oral implantology, maxillofacial surgery and guided-endodontic digital workflows for ensuring a correct diagnosis and accurate virtual treatment planning.^38,39^ Also, it is an indispensable step in the imaging chain allowing successful monitoring of complex maxillofacial reconstructive procedures at follow-up.^40^ If an accurate segmentation is not achieved, then it might negatively influence the further steps in the treatment planning phase. For instance, it could impede an accurate registration with the surface-based data, thereby resulting in an imprecise planning of surgical drilling guides for implant placement and occlusal wafers in orthognathic surgery.^41,42^ The segmentation quality is dependent on the CBCT parameters settings, where a smaller voxel size might improve the image quality with a better segmentation accuracy, however, requiring a higher radiation dose.^33^

Another common shortcoming observed with segmentation of CBCT images using semi- and fully automatic third party software programs is that the thresholding level for the automatic segmentation of these software algorithms has been originally developed for medical CT images with standardized Hounsfield units (HUs), which cannot be applied to CBCT images.^43^ These software packages utilize a sensitivity tool known as “HU” for identifying the density value of a scan and segmenting based on voxel value (also known as grayscale value/gray value) which corresponds to that of the CT HUs and not the voxel values obtained from a CBCT image.^33,44^ For that reason, manual segmentation by an expert could be considered as a solution over thresholding-based segmentation techniques. However, it is a very subjective technique, which relies on the observer’s experience and is also time-consuming. Additionally, when transferring the CBCT data to third-party software programs for segmentation, the data export causes image degradation if data compression is performed using a suboptimal (lossy) method,^33^ which can further influence the segmentation and accuracy of the later steps in the digital workflow. To overcome the information loss during data export, it is advisable to either use the original data for segmentation or to apply lossless compression algorithms when data are being transferred.^45,46^

**Figure 1.**
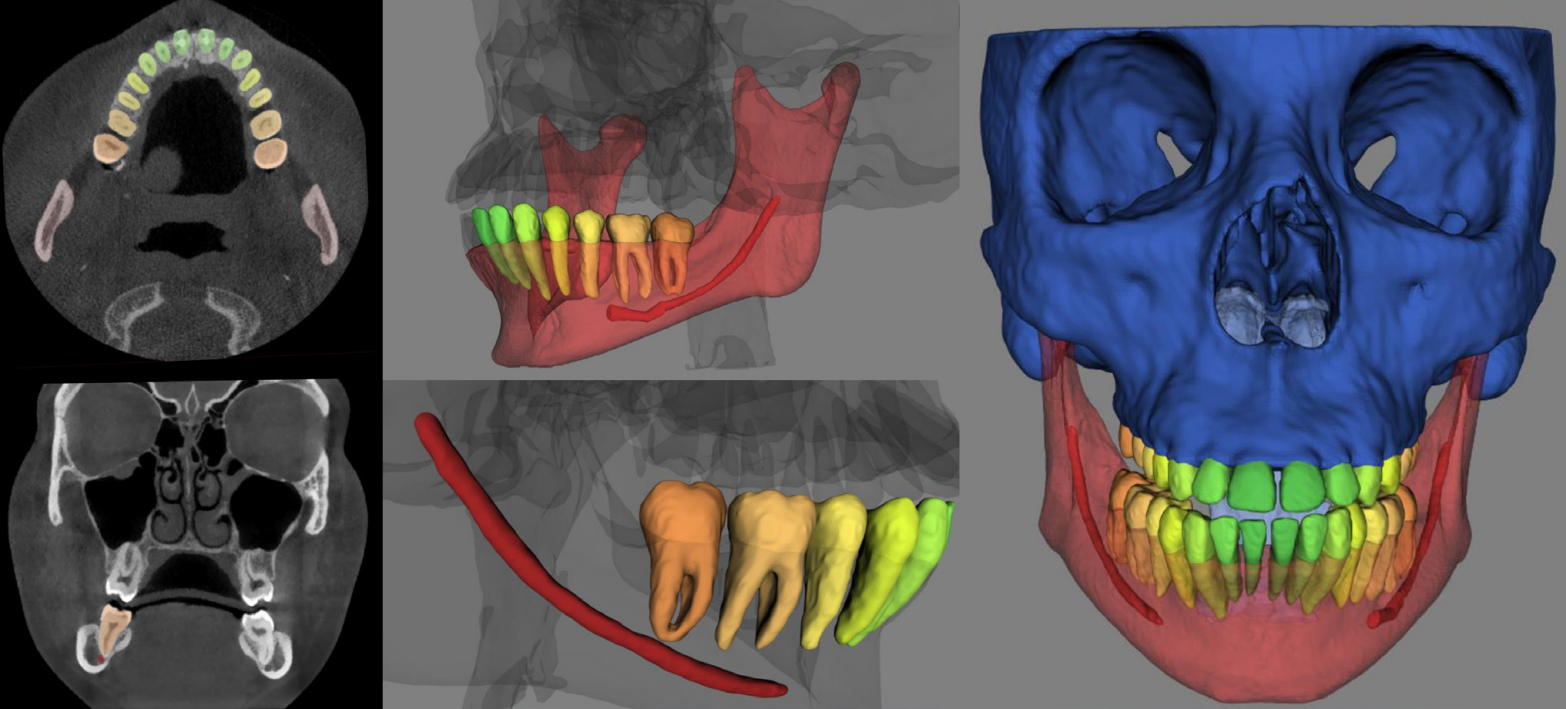
Artificial intelligence-based segmentation of maxillofacial skeletal structures (mandible *vs* facial skeleton), teeth and mandibular canal.

Recently, various artificial intelligence (AI)-based networks have been deployed to overcome errors associated with segmentation and to simplify such digital workflows. Most of these AI-based machine- or deep learning networks have been applied for segmenting the teeth and skeletal structures and have provided methods to precisely segment even in the presence of artifacts.^47–49^ Figure 1 illustrates an example of the dentomaxillofacial structures segmented with an online cloud-based 3D deep learning convolutional neural network platform (v. 1.0, Toothflow, Relu Inc, Leuven, Belgium).

Although, these algorithms have been deemed highly accurate and time-efficient for segmenting anatomical structures in the dentomaxillofacial region, their generalizability is still questionable. Due to the wide variety of CBCT devices offering different scanning parameters and technical settings, all these factors influence the accuracy of AI-based segmentation. For instance, as the AI-network is trained on a specific data set acquired from a single CBCT device, the segmentation outcome might be optimal for only the images acquired with the same device and settings, hence requiring further optimization.

### Intraoral image acquisition

The advancements in CBCT imaging have come a long way since its initial steps in clinical practice to provide accurate bone tissue segmentation. However, it still fails to provide adequate information related to the dentogingival tissue, interocclusal relationship and bite registration.^50,51^ Moreover, the presence of patient motion and artifacts from restorative materials or metal including dental implants further hampers its ability to provide an accurate 3D replica of dentogingival tissue. The conventional workflows which were based on acquiring stone models for the diagnosis and treatment planning in various fields of dentistry offered the limitation of dimensional changes and mould instability due to the ongoing chemical changes within the material.^3^

To overcome the inherent inaccuracies associated with dentogingival tissue capture using CBCT imaging and conventional impression techniques, IOS have streamlined the digital workflows by providing with a more realistic and accurate surface reproduction. Currently, the main clinical applications of IOS are designing of inlays/onlays, veneers and crowns in restorative dentistry, denture frameworks and cleft obturators in prosthodontics, rendering customized archwires, fixed and removable appliance, aligners and indirect bonding trays in orthodontics, guided-surgery in implantology and production of surgical guides and wafers in maxillofacial reconstructive procedures.^52,53^ The optical impressions have offered multiple advantages when incorporated into the digital workflow by offering decreased patient discomfort, time efficiency and better communication with the patient and dental technician.^54^

At the same instance, the disadvantages of IOS cannot be ignored. To date, extensive evidence exists justifying the trueness and precision of intraoral scanning and studies have found it to be clinically satisfactory for various dental procedures.^52,55^ However, most of this evidence has been based on justifying the accuracy for manufacturing short-span prosthesis or restorations and 3D modelling templates.^56^ When considering the accuracy for acquiring a full-arch impression for a long-span prosthesis, conventional impression methods still remain the clinical standard as the IOS error rises with an increase in the edentulous scanning area.^57^ Further research needs to be conducted to improve the precision and trueness of IOS for whole arch scanning and to observe its influence on the accuracy of designing a long-span prosthesis.

### Facial soft tissue image acquisition

A realistic representation of the facial soft tissue is of vital importance for an optimal treatment planning and outcome assessment in the digital workflows of orthodontics and maxillofacial reconstructive surgery. Additionally, its importance in prosthodontic, restorative and dental implantology workflows cannot be denied, where rehabilitation of the aesthetic zone is at question.^58^

To date, CBCT-based images lack the ability to offer a diagnostically distinct 3D image of the soft tissue due to a lack of contrast resolution and texture, which limits its application in the aforementioned digital workflows for soft tissue evaluation.^33^ Also, the application of stabilization aids used for CBCT imaging protocols such as chin rests or forehead restraints might further distort the surface anatomy of the facial soft tissue.^51^ To overcome the provision of the lack of soft tissue information acquired with CBCT devices, 3D scanners have been employed into the digital workflows such as stereophotogrammetry, laser, and structured-light systems, providing a non-ionizing method of image acquisition to create a replica of the facial soft tissue with an accurate representation of the static geometry and texture in three dimensions.^59^ The stereophotogrammetric devices should offer a distinct advantage over the other systems by providing a fast acquisition time, a single shot capture offering a wider coverage of the facial soft tissue of up to 360 degrees and decrease the impact of the facial movements. However, based on current evidence all 3D scanners whether stationary or portable offer the same range of accuracy without any clinically significant difference if scanning protocols are appropriately followed.^60^

Various four-dimensional (4D) stereophotogrammetric devices are also available in the market. 4D imaging which is defined as “‘a time sequence of 3D models of facial animations” allows recording of facial dynamics.^61^ Clinically, these real-time motion imaging devices are mostly applied in the digital workflows of cleft lip and palate and orthognathic surgery for assessing the surgical outcomes in relation to the facial soft tissue symmetry by asking patients to perform certain facial expressions.^62^ At present, the clinical applications of 4D imaging are limited due to its high cost and time-consumption, and is mostly used for research purposes. The current digital workflows rely on the 3D devices as these are faster and clinically more acceptable in the virtual clinical evaluation phase where all steps in the workflow are static. Future studies should focus on generating a real-time 4D virtual patient, where the integration of functional and dynamic records of the soft tissue, muscle tonicity and jaw motion path might allow to establish a comprehensive 4D treatment plan especially for surgical workflows. Additionally, 4D imaging could potentially act as a valuable tool for the smile design process by adding motion to observe the virtual smile path. This would then allow an efficient and dynamic treatment planning including relevant considerations of orofacial aesthetics.^63^

### Registration of images

The final step in the imaging chain of the digital workflows is related to registration or superimposition of the acquired 3D images of the skeletal structures, teeth and soft tissue for the creation of a 3D virtual augmented model.^64^ The image quality of the dentogingival tissue displayed on the CBCT scan and the presence of artifacts makes it difficult to segment the teeth and to plan an accurate dental procedure or design an optimal surgical guide, so it is recommended to replace the region with virtual surface data of the teeth acquired from the IOS.^41^ The integration of the segmented CBCT model with the intraorally scanned teeth is commonly performed by a surface matching method, where the same teeth surfaces or anatomical regions on both the CBCT and IOS are selected to perform the registration. Even while integrating the scanned teeth into the CBCT model, the influence of CBCT artifacts from restorations and orthodontic appliances makes it difficult to achieve a perfect registration.^50^ Some authors have recommended the application of intraoral reference devices with fiducial markers or attachment of titanium markers onto the gingiva to facilitate an accurate registration process which is a time-consuming process.^51,65^ Others have suggested voxel-based registration by acquiring a double scan—a low resolution CBCT of the dentoskeletal region integrated with the teeth acquired from a high resolution CBCT to obtain an augmented virtual model.^66^ The drawback of this method is the violation of the ALARA principle due to the increased radiation dose as well as the increased potential for doubling segmentation errors. For most digital workflows used in dental medicine today, the registration is performed with surface-based registration. This approach uses an iterative closest point (ICP) algorithm for replacing the segmented CBCT dentogingival region with the IOS image. The corresponding surfaces are selected from both images and the algorithm automatically allows superimposition based on the similar shape features.^67^ However, its accuracy is questionable in cases where a CBCT image consists of streak metal artefacts from orthodontics brackets, which has a deleterious effect on the segmentation process and in turn might negatively influence the superimposition accuracy.

Similarly, integration of the facial soft tissue onto the CBCT data is commonly acquired with surface-based registration. The accuracy of the registration is dependent on various patient-related and image acquisition factors, such as, soft tissue and muscular apparatus changes, jaw and head movement, patient positioning and patient stabilization devices. Although, the facial soft tissue surface artifacts induced by the patient movement (swallowing, breathing, head movement) during CBCT capture are minimized when using non-ionizing facial soft tissue imaging devices due to their short acquisition time. However, due to the dissimilarities between the data acquired from both devices, the issue of an accurate registration still persists.^68,69^ At the same instance, if the CBCT scan is not followed immediately by the facial soft tissue capture or vice versa, there is a higher risk of an inaccurate registration due to the changes in the soft tissue drape with a higher potential of patient movement.^51^ Also, it is recommended to acquire the CBCT without a stabilization device to overcome the deformation of the soft tissue for an accurate registration, otherwise the cumulative error might increase and lead to inaccurate treatment planning. One solution to improve the accuracy of the registration could be the simultaneous capture of the CBCT image and soft tissue by placing the facial scanner in front of the patient during CBCT acquisition.^70^ Another alternative could be the utilization of CBCT devices integrated with a facial scanner, allowing the capture of both, CBCT and soft tissue with a single scan.^71^

Lastly, even though with the introduction of state-of-art technologies, an accurate surface registration of the teeth and soft tissue onto the CBCT data is a time-consuming process, prone to error and dependent on the accuracy of the segmentation of dentoskeletal and soft tissue structures from the CBCT image. Recently, AI-based neural network algorithms have been proposed for the automatic fusion of the intraoral scan and CBCT images without any human intervention.^72^ However, further research needs to be conducted to refine their accuracy.

## Conclusions and future outlook

Imaging forms the basis of all the digital workflows in dentistry. The accumulation of errors at every step of the workflow might negatively influence the treatment planning, final outcome and monitoring at follow-up. Therefore, it is important to assess the induced collective error and how it might influence the clinical efficacy of a workflow. For an efficient digital workflow, it is important to be aware of the existing challenges within the imaging chain and come up with strategies for making the workflows more simplified and efficient. Although recent technological advancements have drastically enhanced the imaging aspect of the digital workflows, there is still room for further improvement by integrating AI-based strategies. The following future possibilities should be considered to further simplify the imaging workflow and to facilitate a personalized and predictive treatment planning and outcome assessment;

- Development of AI-based neural-network algorithms for automatic segmentation of dentomaxillofacial structures an improved virtual diagnosis and treatment planning.
- Generalization of the AI segmentation techniques by standardizing scanning. parameters used, propose and agree on patient- and workflow-specific guidelines, and train AI algorithms with larger data sets to improve the precision of the segmentation in the digital workflows..
- Development of an automated AI-based fusion algorithm for registration of the facial soft tissue and teeth surface data onto the segmented skull without any manual intervention or dependency on the selection of the region of interest.
- Application of imaging-based AI algorithms for the prediction of treatment outcomes and automatic assessment of tissue healing, growth and follow-up changes.

**Figure 2.**
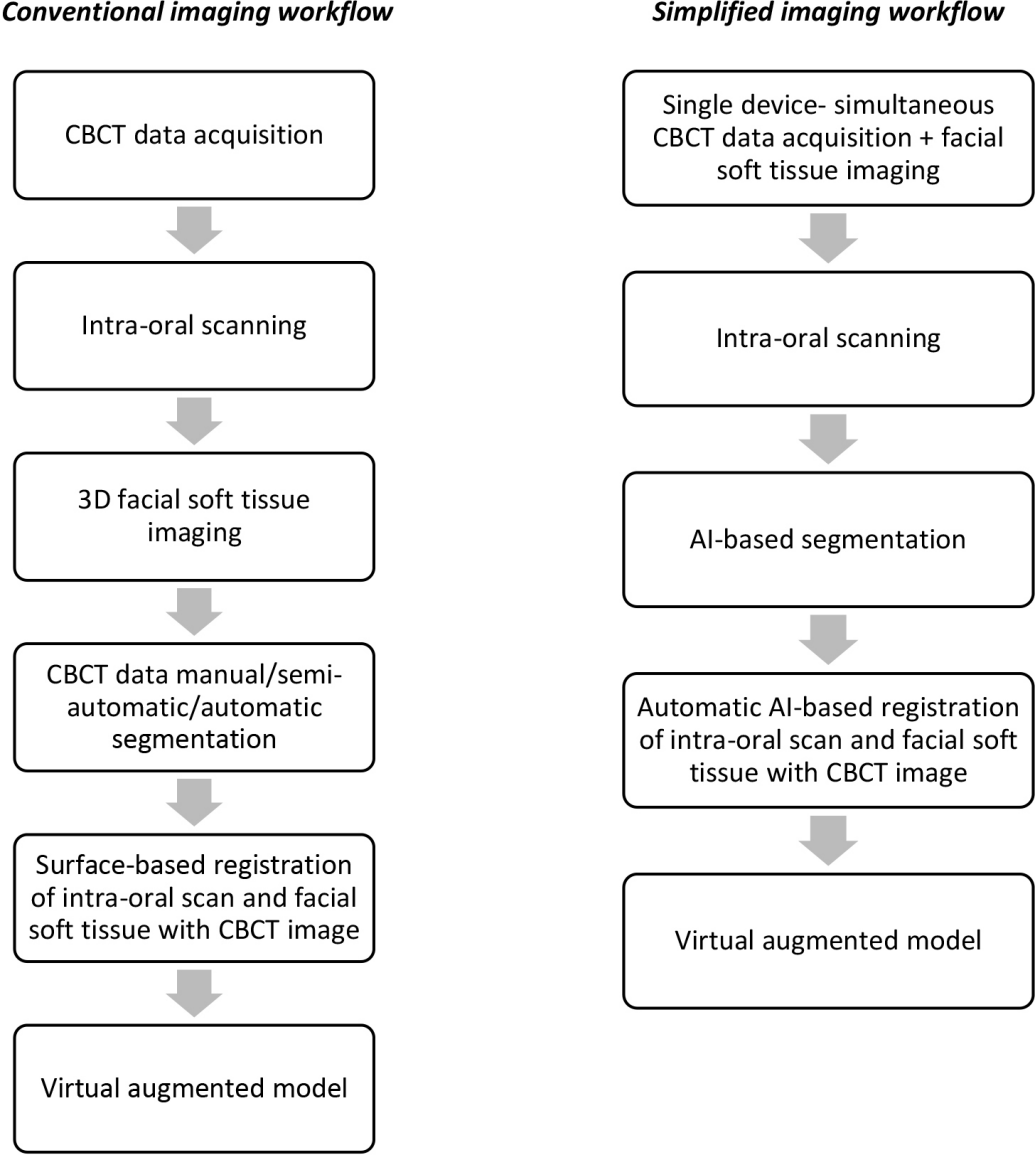
Steps of workflow in a conventional digital imaging chain and recommended improvements based on induction of artificial intelligence based systems to simply and improve the accuracy of workflow. AI, artificial intelligence; CBCT, cone-beam computed tomography

The application of these networks and state-of-art technologies will definitely add to the time-efficiency of present digital workflows and could become a clinical standard in the near future for workflows where integration of 3D images forms the basis of treatment planning and outcome assessment (Figure 2).

## References

[1] Rekow ED. Digital dentistry: The new state of the art - Is it disruptive or destructive? Dent Mater 2020; 36: 9–24. doi: 10.1016/j.dental.2019.08.103 31526522

[2] Joda T, Bornstein MM, Jung RE, Ferrari M, Waltimo T, Zitzmann NU. Recent trends and future direction of dental research in the digital era. Int J Environ Res Public Health 2020; 17: 1–8. doi: 10.3390/ijerph17061987 32197311 PMC7143449

[3] Lin Y-M. Digitalisation in Dentistry: Development and Practices. : The digitization of business in China: Springer International Publishing; 2018. . 199–217.

[4] Mangano C, Luongo F, Migliario M, Mortellaro C, Mangano FG. Combining intraoral scans, cone beam computed tomography and face scans: the virtual patient. J Craniofac Surg 2018; 29: 2241–6. doi: 10.1097/SCS.0000000000004485 29698362

[5] Vandenberghe B. The digital patient - Imaging science in dentistry. J Dent 2018; 74 Suppl 1: S21–6. doi: 10.1016/j.jdent.2018.04.019 29929585

[6] Joda T, Ferrari M, Gallucci GO, Wittneben J-G, Brägger U. Digital technology in fixed implant prosthodontics. Periodontol 2000 2017; 73: 178–92. doi: 10.1111/prd.12164 28000274

[7] Cervino G, Fiorillo L, Arzukanyan A, Spagnuolo G, Cicciù M. Dental restorative digital workflow: digital SMILE design from aesthetic to function. Dent J 2019; 7: 30. doi: 10.3390/dj7020030 PMC6632039 30925698

[8] Hosaka K, Tichy A, Motoyama Y, Mizutani K, Lai W-J, Kanno Z, et al. Post-orthodontic recontouring of anterior teeth using composite injection technique with a digital workflow. J Esthet Restor Dent 2020; 32: 638–44. doi: 10.1111/jerd.12619 32603555

[9] da Silva Salomão GV, Chun EP, Panegaci RDS, Santos FT. Analysis of digital workflow in Implantology. Case Rep Dent 2021; 2021: 1–7. doi: 10.1155/2021/6655908 33628525 PMC7899756

[10] Christensen LR. Digital workflows in contemporary orthodontics. APOS 2017; 7: 12–18. doi: 10.4103/2321-1407.199180

[11] Stanley M, Paz AG, Miguel I, Coachman C. Fully digital workflow, integrating dental scan, SMILE design and CAD-CAM: case report. BMC Oral Health 2018; 18: 134. doi: 10.1186/s12903-018-0597-0 30086753 PMC6081948

[12] Park J-S, Lim Y-J, Kim B, Kim M-J, Kwon H-B. Clinical evaluation of time efficiency and fit accuracy of lithium disilicate single Crowns between conventional and digital impression. Materials 2020; 13: 1–18. doi: 10.3390/ma13235467 33266314 PMC7730557

[13] Clark WA, Brazile B, Matthews D, Solares J, De Kok IJ. A comparison of conventionally versus digitally fabricated denture outcomes in a university dental clinic. J Prosthodont 2021; 30: 47–50. doi: 10.1111/jopr.13273 33058337

[14] Lin C-C, Wu C-Z, Huang M-S, Huang C-F, Cheng H-C, Wang DP. Fully digital workflow for planning static guided implant surgery: a prospective accuracy study. J Clin Med 2020; 9: 980. doi: 10.3390/jcm9040980 32244735 PMC7231012

[15] Ray JJ, Giacomino CM, Wealleans JA, Sheridan RR. Targeted endodontic microsurgery: digital workflow options. J Endod 2020; 46: 863–71. doi: 10.1016/j.joen.2020.02.006 32284239

[16] Van der Groen T, Lee K, Archer B. Eliminating stone models in orthognathic surgery: a complete digital workflow. Journal of Oral and Maxillofacial Surgery 2020; 78: e79. doi: 10.1016/j.joms.2020.07.156

[17] Block MS, Emery RW, Cullum DR, Sheikh A. Implant placement is more accurate using dynamic navigation. J Oral Maxillofac Surg 2017; 75: 1377–86. doi: 10.1016/j.joms.2017.02.026 28384461

[18] Dianat O, Nosrat A, Tordik PA, Aldahmash SA, Romberg E, Price JB, et al. Accuracy and efficiency of a dynamic navigation system for locating calcified canals. J Endod 2020; 46: 1719–25. doi: 10.1016/j.joen.2020.07.014 32692993

[19] Vandenberghe B. The crucial role of imaging in digital dentistry. Dent Mater 2020; 36: 581–91. doi: 10.1016/j.dental.2020.03.001 32299666

[20] Joda T, Yeung AWK, Hung K, Zitzmann NU, Bornstein MM. Disruptive innovation in dentistry: what it is and what could be next. J Dent Res 2021; 100: 448–53. doi: 10.1177/0022034520978774 33322997

[21] Lamoral Y, Quirynen M, Peene P, Vanneste F, Lemahieu SF, Baert AL, et al. Computed tomography in the preoperative planning of oral endo-osseous implant surgery. Rofo 1990; 153: 505–9. doi: 10.1055/s-2008-1033428 2173053

[22] Quirynen M, Lamoral Y, Dekeyser C, Peene P, van Steenberghe D, Bonte J, et al. Ct scan standard reconstruction technique for reliable jaw bone volume determination. Int J Oral Maxillofac Implants 1990; 5: 384–9. 2094657

[23] Schwarz MS, Rothman SL, Rhodes ML, Chafetz N. Computed tomography: Part I. preoperative assessment of the mandible for endosseous implant surgery. Int J Oral Maxillofac Implants 1987; 2: 137–741. 3481354

[24] Schwarz MS, Rothman SL, Rhodes ML, Chafetz N. Computed tomography: Part II. preoperative assessment of the maxilla for endosseous implant surgery. Int J Oral Maxillofac Implants 1987; 2: 143–8. 3481355

[25] Zhao L, Patel PK, Cohen M. Application of virtual surgical planning with computer assisted design and manufacturing technology to cranio-maxillofacial surgery. Arch Plast Surg 2012; 39: 309–16. doi: 10.5999/aps.2012.39.4.309 22872832 PMC3408274

[26] Mozzo P, Procacci C, Tacconi A, Martini PT, Andreis IA, Tinazzi Martini P, Bergamo Andreis IA. A new volumetric CT machine for dental imaging based on the cone-beam technique: preliminary results. Eur Radiol 1998; 8: 1558–64. doi: 10.1007/s003300050586 9866761

[27] Kusnoto B, Kaur P, Salem A, Zhang Z, Galang-Boquiren MT, Viana G, et al. Implementation of ultra-low-dose CBCT for routine 2D orthodontic diagnostic radiographs: cephalometric landmark identification and image quality assessment. Semin Orthod 2015; 21: 233–47. doi: 10.1053/j.sodo.2015.07.001

[28] Lee HC, Song B, Kim JS, Jung JJ, Li HH, Mutic S, et al. An efficient iterative CBCT reconstruction approach using gradient projection sparse reconstruction algorithm. Oncotarget 2016; 7: 87342–50. doi: 10.18632/oncotarget.13567 27894103 PMC5349992

[29] Yeung AWK, Jacobs R, Bornstein MM. Novel low-dose protocols using cone beam computed tomography in dental medicine: a review focusing on indications, limitations, and future possibilities. Clin Oral Investig 2019; 23: 2573–81. doi: 10.1007/s00784-019-02907-y 31025192

[30] Gaêta-Araujo H, Alzoubi T, Vasconcelos KdeF, Orhan K, Pauwels R, Casselman JW, et al. Cone beam computed tomography in dentomaxillofacial radiology: a two-decade overview. Dentomaxillofac Radiol 2020; 49: 20200145. doi: 10.1259/dmfr.20200145 32501720 PMC7719864

[31] Jacobs R, Quirynen M. Dental cone beam computed tomography: justification for use in planning oral implant placement. Periodontol 2000 2014; 66: 203–13. doi: 10.1111/prd.12051 25123769

[32] Bornstein MM, Scarfe WC, Vaughn VM, Jacobs R. Cone beam computed tomography in implant dentistry: a systematic review focusing on guidelines, indications, and radiation dose risks. Int J Oral Maxillofac Implants 2014; 29(Suppl): 55–77. doi: 10.11607/jomi.2014suppl.g1.4 24660190

[33] Jacobs R, Salmon B, Codari M, Hassan B, Bornstein MM. Cone beam computed tomography in implant dentistry: recommendations for clinical use. BMC Oral Health 2018; 18: 88. doi: 10.1186/s12903-018-0523-5 29764458 PMC5952365

[34] Gaêta-Araujo H, Leite AF, Vasconcelos KdeF, Jacobs R. Two decades of research on CBCT imaging in DMFR - an appraisal of scientific evidence. Dentomaxillofac Radiol 2021; 50: 50. doi: 10.1259/dmfr.20200367 33555198 PMC8077993

[35] Oenning AC, Jacobs R, Pauwels R, Stratis A, Hedesiu M, Salmon B, et al. Cone-Beam CT in paediatric dentistry: DIMITRA project position statement. Pediatr Radiol 2018; 48: 308–16. doi: 10.1007/s00247-017-4012-9 29143199

[36] Nomura Y, Watanabe H, Manila NG, Asai S, Kurabayashi T. Evaluation of streak metal artifacts in cone beam computed tomography by using the Gumbel distribution: a phantom study. Oral Surg Oral Med Oral Pathol Oral Radiol 2021; 131: 494–502. doi: 10.1016/j.oooo.2020.08.031 33020029

[37] Pauwels R, Araki K, Siewerdsen JH, Thongvigitmanee SS. Technical aspects of dental CBCT: state of the art. Dentomaxillofac Radiol 2015; 44: 20140224. doi: 10.1259/dmfr.20140224 25263643 PMC4277439

[38] Wang H, Minnema J, Batenburg KJ, Forouzanfar T, Hu FJ, Wu G. Multiclass CBCT image segmentation for orthodontics with deep learning. J Dent Res 2021; 100: 943–9. doi: 10.1177/00220345211005338 33783247 PMC8293763

[39] Antila K, Lilja M, Kalke M. Segmentation of facial bone surfaces by patch growing from cone beam CT volumes. Dentomaxillofac Radiol 2016; 45: 20150435. doi: 10.1259/dmfr.20150435 27482878 PMC5595020

[40] Vaitiekūnas M, Jegelevičius D, Sakalauskas A, Grybauskas S. Automatic method for bone segmentation in cone beam computed tomography data set. Appl Sci 2020; 10: 236. doi: 10.3390/app10010236

[41] Flügge T, Derksen W, Te Poel J, Hassan B, Nelson K, Wismeijer D. Registration of cone beam computed tomography data and intraoral surface scans - A prerequisite for guided implant surgery with CAD/CAM drilling guides. Clin Oral Implants Res 2017; 28: 1113–8. doi: 10.1111/clr.12925 27440381 PMC5599947

[42] Almutairi T, Naudi K, Nairn N, Ju X, Whitters J, Ayoub A. Replacement of the distorted dentition of the cone-beam computed tomography scans for orthognathic surgery planning. J Oral Maxillofac Surg 2018; 76: 1561.e1–1561.e8. doi: 10.1016/j.joms.2018.02.018 29572134

[43] Pauwels R, Jacobs R, Singer SR, Mupparapu M. CBCT-based bone quality assessment: are Hounsfield units applicable? Dentomaxillofac Radiol 2015; 44: 20140238. doi: 10.1259/dmfr.20140238 25315442 PMC4277442

[44] van Eijnatten M, Koivisto J, Karhu K, Forouzanfar T, Wolff J. The impact of manual threshold selection in medical additive manufacturing. Int J Comput Assist Radiol Surg 2017; 12: 607–15. doi: 10.1007/s11548-016-1490-4 27718124 PMC5362669

[45] Graham RNJ, Perriss RW, Scarsbrook AF. DICOM demystified: a review of digital file formats and their use in radiological practice. Clin Radiol 2005; 60: 1133–40. doi: 10.1016/j.crad.2005.07.003 16223609

[46] Yan H, Li Y, Dai J. Evaluation of video compression methods for cone-beam computerized tomography. J Appl Clin Med Phys 2019; 20: 114–21. doi: 10.1002/acm2.12596 31074197 PMC6753726

[47] Hung K, Montalvao C, Tanaka R, Kawai T, Bornstein MM. The use and performance of artificial intelligence applications in dental and maxillofacial radiology: a systematic review. Dentomaxillofac Radiol 2020; 49: 49. doi: 10.1259/dmfr.20190107 31386555 PMC6957072

[48] Tuzoff DV, Tuzova LN, Bornstein MM, Krasnov AS, Kharchenko MA, Nikolenko SI, et al. Tooth detection and numbering in panoramic radiographs using convolutional neural networks. Dentomaxillofac Radiol 2019; 48: 20180051. doi: 10.1259/dmfr.20180051 30835551 PMC6592580

[49] Lahoud P, EzEldeen M, Beznik T, Willems H, Leite A, Van Gerven A. Artificial intelligence for fast and accurate 3D tooth segmentation on CBCT. J Endod 2021; 47: 827–35. doi: 10.1016/j.joen.2020.12.020 33434565

[50] Rangel FA, Maal TJJ, de Koning MJJ, Bronkhorst EM, Bergé SJ, Kuijpers-Jagtman AM. Integration of digital dental casts in cone beam computed tomography scans-a clinical validation study. Clin Oral Investig 2018; 22: 1215–22. doi: 10.1007/s00784-017-2203-2 28932947 PMC5866842

[51] Elnagar MH, Aronovich S, Kusnoto B. Digital workflow for combined orthodontics and orthognathic surgery. Oral Maxillofac Surg Clin North Am 2020; 32: 1–14. doi: 10.1016/j.coms.2019.08.004 31699582

[52] Mangano F, Gandolfi A, Luongo G, Logozzo S. Intraoral scanners in dentistry: a review of the current literature. BMC Oral Health 2017; 17: 149. doi: 10.1186/s12903-017-0442-x 29233132 PMC5727697

[53] Dalbah L. Digital Orthodontics. In: Digitization in dentistry; 2021. pp. 189–221.

[54] Cicciù M, Fiorillo L, D'Amico C, Gambino D, Amantia EM, Laino L, et al. 3D digital impression systems compared with traditional techniques in dentistry: a recent data systematic review. Materials 2020; 13: E1982. doi: 10.3390/ma13081982 32340384 PMC7215909

[55] Koch GK, Gallucci GO, Lee SJ. Accuracy in the digital workflow: from data acquisition to the digitally milled cast. J Prosthet Dent 2016; 115: 749–54. doi: 10.1016/j.prosdent.2015.12.004 26803173

[56] Ebeid K, Salah T, Nossair S. Accuracy and reliability of intraoral scanners: are they the better option? Curr Oral Health Rep 2017; 4: 209–14. doi: 10.1007/s40496-017-0145-z

[57] Suese K. Progress in digital dentistry: the practical use of intraoral scanners. Dent Mater J 2020; 39: 52–6. doi: 10.4012/dmj.2019-224 31723066

[58] Hassan B, Greven M, Wismeijer D. Integrating 3D facial scanning in a digital workflow to CAD/CAM design and fabricate complete dentures for immediate total mouth rehabilitation. J Adv Prosthodont 2017; 9: 381–6. doi: 10.4047/jap.2017.9.5.381 29142646 PMC5673615

[59] Mai HN, Kim J, Choi YH, Lee DH. Accuracy of portable face-scanning devices for obtaining three-dimensional face models: a systematic review and meta-analysis. Int J Environ Res Public Health 2021; 18: 1–15. doi: 10.3390/ijerph18010094 PMC7795319 33375533

[60] Heike CL, Upson K, Stuhaug E, Weinberg SM. 3D digital stereophotogrammetry: a practical guide to facial image acquisition. Head Face Med 2010; 6: 18. doi: 10.1186/1746-160X-6-18 20667081 PMC2920242

[61] Shujaat S, Khambay BS, Ju X, Devine JC, McMahon JD, Wales C, et al. The clinical application of three-dimensional motion capture (4D): a novel approach to quantify the dynamics of facial animations. Int J Oral Maxillofac Surg 2014; 43: 907–16. doi: 10.1016/j.ijom.2014.01.010 24583138

[62] Erten O, Yılmaz BN. Three-Dimensional imaging in Orthodontics. Turk J Orthod 2018; 31: 86–94. doi: 10.5152/TurkJOrthod.2018.17041 30206567 PMC6124883

[63] Jreige CS, Kimura RN, Segundo Ângelo Raphael Toste Coelho, Coachman C, Sesma N. Esthetic treatment planning with digital animation of the SMILE dynamics: a technique to create a 4-dimensional virtual patient. J Prosthet Dent 2021;09 Feb 2021. doi: 10.1016/j.prosdent.2020.10.015 33573832

[64] Joda T, Brägger U, Gallucci G. Systematic literature review of digital three-dimensional superimposition techniques to create virtual dental patients. Int J Oral Maxillofac Implants 2015; 30: 330–7. doi: 10.11607/jomi.3852 25830393

[65] Yang W-M, Ho C-T, Lo L-J. Automatic superimposition of palatal fiducial markers for accurate integration of digital dental model and cone beam computed tomography. J Oral Maxillofac Surg 2015; 73: 1616.e1–1616.e10. doi: 10.1016/j.joms.2015.04.004 25957873

[66] Swennen GRJ, Mollemans W, De Clercq C, Abeloos J, Lamoral P, Lippens F, et al. A cone-beam computed tomography triple scan procedure to obtain a three-dimensional augmented virtual skull model appropriate for orthognathic surgery planning. J Craniofac Surg 2009; 20: 297–307. doi: 10.1097/SCS.0b013e3181996803 19276829

[67] Jung K, Jung S, Hwang I, Kim T, Chang M. Registration of dental tomographic volume data and scan surface data using dynamic segmentation. Appl Sci 2018; 8: 1762. doi: 10.3390/app8101762

[68] Schendel SA, Lane C. 3D orthognathic surgery simulation using image fusion. Semin Orthod 2009; 15: 48–56. doi: 10.1053/j.sodo.2008.09.012

[69] Nahm K-Y, Kim Y, Choi Y-S, Lee J, Kim S-H, Nelson G. Accurate registration of cone-beam computed tomography scans to 3-dimensional facial Photographs. Am J Orthod Dentofacial Orthop 2014; 145: 256–64. doi: 10.1016/j.ajodo.2013.10.018 24485741

[70] Naudi KB, Benramadan R, Brocklebank L, Ju X, Khambay B, Ayoub A. The virtual human face: superimposing the simultaneously captured 3D photorealistic skin surface of the face on the untextured skin image of the CBCT scan. Int J Oral Maxillofac Surg 2013; 42: 393–400. doi: 10.1016/j.ijom.2012.10.032 23228692

[71] Rasteau S, Sigaux N, Louvrier A, Bouletreau P. Three-dimensional acquisition technologies for facial soft tissues - Applications and prospects in orthognathic surgery. J Stomatol Oral Maxillofac Surg 2020; 121: 721–8. doi: 10.1016/j.jormas.2020.05.013 32442635

[72] Chung M, Lee J, Song W, Song Y, Yang I-H, Lee J, et al. Automatic registration between dental cone-beam CT and scanned surface via deep pose regression neural networks and clustered similarities. IEEE Trans Med Imaging 2020; 39: 3900–9. doi: 10.1109/TMI.2020.3007520 32746134

